# Clinical implications of differences between real world and clinical trial usage of left ventricular assist devices for end stage heart failure

**DOI:** 10.1371/journal.pone.0242928

**Published:** 2020-12-03

**Authors:** Catherine Mezzacappa, Neal G. Ravindra, Cesar Caraballo, Fouad Chouairi, P. Elliott Miller, John-Ross D. Clarke, Jadry Gruen, Makoto Mori, Megan McCullough, Clancy Mullan, Arnar Geirsson, Joseph G. Rogers, Mohammad Anwer, Nihar Desai, Tariq Ahmad

**Affiliations:** 1 Department of Internal Medicine, Yale University School of Medicine, New Haven, CT, United States of America; 2 Section of Cardiovascular Medicine, Yale University School of Medicine, New Haven, CT, United States of America; 3 Center for Outcome Research & Evaluation (CORE), Yale University School of Medicine, New Haven, CT, United States of America; 4 Section of Cardiothoracic Surgery, Yale University School of Medicine, New Haven, CT, United States of America; 5 Division of Cardiology, Duke University School of Medicine, Durham, NC, United States of America; Thomas Jefferson University, UNITED STATES

## Abstract

**Importance:**

Patient outcomes in heart failure clinical trials are generally better than those observed in real-world settings. This may be related to stricter inclusion and exclusion criteria in clinical trials.

**Objective:**

We study sought to characterize the clinical implications of differences between patients in clinical trials and those in a real-world registry of patients receiving left ventricular assist devices (LVADs).

**Design, setting, and participants:**

This retrospective cohort study included all patients in INTERMACS (the Interagency Registry for Mechanically Assisted Circulatory Support) who were implanted with an axial flow LVAD from 2010 to 2015 to allow for equivalent comparisons.

**Main outcomes and measures:**

Differences in patient characteristics and 2-year rates of adverse outcomes with those reported in the ENDURANCE and MOMENTUM 3 clinical trials. Survival analyses were used to assess the relationships between prespecified patient factors and clinical outcomes.

**Results:**

Of the 10,937 LVAD recipients identified in INTERMACS between 2010–2015, 44% met at least 1 clinical trial exclusion criterion. The 2-year incidence of stroke and death amongst LVAD recipients in INTERMACS and the landmark clinical trials differed significantly (*P*<0.04, both). Nevertheless, patients who would have been excluded from the clinical trials did not have dramatically different 2-year mortality outcomes in INTERMACS [2y survival estimate: 66.4%, 95% CI (64.9–67.9%) versus 71.9%, 95% CI (70.6–73.1%)]. Clinical interventions driving a significantly increased risk of death were relatively rare (<5% of implants) and included mechanical ventilation, ECMO, severe thrombocytopenia, and dialysis.

**Conclusions and relevance:**

Most exclusion criteria used in LVAD clinical trials did not afford a substantially greater risk to patients in the real-world setting. In the relatively infrequent cases of end stage renal disease, thrombocytopenia, respiratory failure, and need for ECMO, the risks and benefits of LVAD therapy need careful weighting and further study.

## Introduction

The dire prognosis of patients with end stage heart failure (HF) has prompted an upsurge in the number of pharmacological and device therapies that are being evaluated for the potential to improve morbidity and mortality [1]. One key success has been the development and subsequent proliferation of durable left ventricular assist devices (LVADs) that offer a therapeutic alternative to heart transplantation [2]. The last few decades have seen substantial progress in LVAD technologies, including revolutions in pump design, miniaturization, and enhanced durability, all of which have led to significant improvements in patient outcomes [3].

Two landmark randomized clinical trials, ENDURANCE and MOMENTUM 3, compared centrifugal flow (study device) to axial flow LVADs [4, 5]. The ENDURANCE trial enrolled patients deemed ineligible for cardiac transplant and demonstrated non-inferiority for a primary endpoint of 2-year survival free from disabling stroke or device removal, as well as less frequent device failure and strokes in the centrifugal flow device group. The MOMENTUM 3 trial enrolled patients with advanced heart failure regardless of transplant eligibility and demonstrated superior 2-year survival free from disabling stroke or device removal, as well as less frequent pump replacement, stroke, and bleeding events in the centrifugal flow device group.

These clinical trials were designed to maximize the probability of successfully demonstrating the benefit of new interventions over standard therapy [4, 5]. As a result, many patients with advanced HF and comorbid conditions, who qualify for the post approval clinical use of LVADs, were excluded from the trials. To determine any divergences between clinical trial data and real-world outcomes, a post FDA device approval registry called Interagency Registry for Mechanically Assisted Circulatory Support (INTERMACS) was created as a joint effort between the National Heart, Lung, and Blood Institute, the Food and Drug Administration, and the Centers for Medicare & Medicaid Services; data is available for analysis until 2017 [6].

In order to examine the differences between real world patients receiving LVADs and those who were enrolled in landmark clinical trials, we compared differences between patient characteristics in two landmark clinical trials and those in the INTERMACS registry of patients. In order to have adequate numbers for the comparison, we focused on those receiving axial flow LVADs in whom there was 2 years of follow up, to mirror findings from the clinical trials analyzed. Finally, we examined rates of adverse clinical outcomes among patients who received LVADS but might not have qualified for the landmark clinical trials.

## Methods

### Data sources

To capture 2 years of post-implantation data, patients in the Interagency Registry for Mechanically Assisted Circulatory Support (INTERMACS) registry who received an FDA-approved durable axial flow mechanical circulatory support device between the first quarter of 2010 and the end of the third quarter of 2015 were included; only data up to 2017 is available for analysis, as the NIH funding for INTERMACS was stopped at that point. As described elsewhere, INTERMACS is a public-private partnership between the NHLBI, the FDA, Centers for Medicaid and Medicare Services, hospitals, and industry[6]. Publicly available data on previously completed definitive clinical trials of LVADs were used. This is available at https://biolincc.nhlbi.nih.gov). The study was approved by the Yale IRB.

#### Real-world patients: INTERMACS

Analysis of the INTERMACS registry was performed for all patients who received a first axial flow LVAD between the 2010 and 2015. We only included axial flow devices to minimize confounding by device type. Since the HeartMate II received FDA approval for use in long-term support or destination therapy in January of 2010, significantly altering the patient population eligible to receive this treatment, we did not include patients who received the device prior to 2010. Patients who received a device after the third quarter of 2015 were not included due to insufficient follow up time to evaluate the outcomes of interest at 2 years post-implantation. Patients who received a biventricular assist device at time of implantation, right ventricular assist device only, total artificial heart, pulsatile device, or centrifugal flow device were excluded from the analysis, as were patients who had a prior LVAD, total artificial heart, or heart transplant. In total, 10,937 patients were included in the study sample. Of note, patients who were enrolled in clinical trials were not included in the INTERMACS dataset.

#### Clinical trial population

Data from the following landmark randomized controlled clinical trials of continuous flow LVADs was used; for this study we only included patients who received an axial flow device to minimize confounding by device type.

1. The ENDURANCE Trial [5]: This trial enrolled from August 2010 to May 2012. It included 297 patients who were randomized to a centrifugal flow device and 148 assigned to the control device (axial flow). Major exclusion criteria included BMI>40 kg/m^2^, use of invasive mechanical ventilation, Platelet count<75,000, INR>2, Creatinine >3.0mg/dL or renal replacement therapy, abnormal elevations in ALT/AST (3 times the ULN), bilirubin >3mg/dL, use of temporary mechanical circulatory support (other than intra-aortic balloon pump), and severe COPD. The primary end-point was survival at 2 years free from disabling stroke or device removal for malfunction or failure.
2. The MOMENTUM 3 Trial [7]: This trial enrolled from September 2014 to November 2015. It included 516 patients randomized to a centrifugal flow device and 512 to an axial flow device. Major exclusion criteria included a platelet count of <100,000/mL, INR≥2.0, bilirubin >2.5mg/dL, severe COPD, creatinine>2.5mg/dL, and albumin<3g/dL. The composite primary end-point was survival at 2 years free of disabling stroke or survival free of reoperation to replace or remove a malfunctioning device.

#### Patient characteristics and endpoints

Patient baseline demographic and health characteristics were ascertained from the Demographics, Pre-Implant, and Implant data collection forms in the INTERMACS patient registry. Patient characteristics mirroring clinical trial exclusion criteria were ascertained from the Pre-Implant form, which reflects the time closest to implant prior to surgery and includes height and weight, IV inotrope use, interventions within 48 hours of implant (mechanical ventilation, ECMO, IABP, dialysis), and laboratory values nearest time of implant. Device information was ascertained from the Implant data collection form in the INTERMACS patient registry. The primary end points were death, stroke, and reoperation to replace or remove a device. Cardiac transplant was treated as a treatment success and right-censored unless it was performed emergently due to device malfunction, in which case it was considered a reoperation treatment failure within the outcome described above. Secondary end points included the cumulative incidence over 2 years post-implantation of adverse events such as infection, bleeding, right heart failure, renal dysfunction, respiratory failure, and LVAD thrombosis.

### Statistical analyses

Baseline patient characteristics were described using means and standard deviations or proportions. For continuous variables, differences were compared using ANOVA and Kruskal-Wallis tests. Categorical variables were compared using ꭕ^2^ and exact tests. ꭕ^2^ goodness-of-fit tests were performed on categorical variables after adjusting the expected proportion for the number of patients in each trial and independent, one-way ANOVA tests were performed on numerical data for summary statistics reported in each trial. Frequencies of clinical endpoints over a 2-year period post-implant, as well 2-year frequencies for common adverse events, were calculated. Kaplan-Meier estimates to assess the relationships between prespecified patient factors and death were performed. Patients who received a heart transplant were censored at the time of transplant. Survival functions comparing overall survival across key patient demographic and clinical criteria that were used to determine eligibility for participation in major trials of LVADs were generated. Cox proportional hazard regression of 2-year mortality including all captured exclusion criteria was performed. All data analysis was conducted in R version 3.6.1 (R Foundation for Statistical Computing, Vienna, Austria).

## Results

### Baseline characteristics

Baseline characteristics of patients in INTERMACS who received an axial flow device are shown in **Table 1**, alongside patients with this device who were enrolled in landmark clinical trials. Patients overall tended to be older (mean age: 58–66), white (69–78%), and male (80–82%). The prevalence of ischemic cardiomyopathy and prior stroke was higher in the trials versus registry (*P*<0.001, both). Use of heart failure therapies were broadly similar across the strata; ICD use was lowest among MOMENTUM and highest in ENDURANCE patients. A significantly greater percentage of patients were classified as having critical cardiogenic shock (INTERMACS Profile 1) and inotrope dependence (INTERMACS Profile 3) in the registry versus clinical trials (*P*<0.001, both). Of note, a comprehensive comparison of variables was limited by non-availability of some patient variables from publicly available data on clinical trials. Furthermore, subjective patient classifications (e.g. INTERMACS profile) likely differed considerably between clinical trials and the INTERMACS registry due to differences in methodology of assessment.

**Table 1:** Baseline characteristics of patients in the INTERMACS registry and in landmark clinical trials who received durable axial flow LVADs.

Patient Characteristics	HM II LVAD	INTERMACS	HM II LVAD Recipients	*P*
	Recipients (MOMENTUM)	Axial Flow LVADs		
			(ENDURANCE)	
**Time Frame**	2014–2019	2010–2015	2010–2014	
**Number**	512	10,937	148	
Age, Years	60 (12)	58.0 (12.9)	66.2 (10.2)	<0.001
Male, %	81.8	79.5	82.4	0.31
White, %	71.7	68.8	77.7	0.03
BSA	2.1 (0.3)	2.1 (0.3)	2.0 (0.3)	<0.001
Coronary Artery Bypass, %	22.3	23.3	N/A	0.63
Valve Replacement/Repair, %	6.1	2.8	N/A	<0.001
Ischemic Cardiomyopathy, %	46.9	42.5	60.1	<0.001
Stroke*, %	10.9	3.9	16.2	<0.001
Inotropes, %	82.6	80.7	73.0	0.03
Diuretics, %	90.8	90.4	81.8	0.002
ACE Inhibitor or ARB, %	33.8	36.9	27.7^†^	0.03
Beta-Blockers, %	53.3	55.2	57.4	0.60
Sodium	135.5 (4.2)	135.0 (4.7)	134.8 (4.8)	0.05
Creatinine	1.4 (0.4)	1.4 (0.7)	1.4 (0.5)	1.00
ICD, %	74.6	81.5	91.2	<0.001
Critical Cardiogenic Shock (IM1), %	3.5	13.5	3.4	<0.001
Progressive Decline (IM2), %	28.5	36.0	31.1	0.001
Stable but inotrope dependent (IM3), %	49.0	31.8	40.5	<0.001
Resting Symptoms (IM4). %	16.0	14.5	18.2	0.29
Exertion intolerant, limited, or Advanced NYHA Class III (IM 5–7), %	2.9^‡^	4.2	6.8	0.11

*In INTERMACS, this is limited to provider indication of history of major stroke in the patient as a barrier to transplantation. In the ENDURANCE trial, it included stroke and TIA. In INTERMACS, *N* = 6981 for 2010–2015 and there was insufficient data to report for 2008–2009.

^†^In the ENDURANCE trial only number of patients on ACEi was reported.

^‡^INTERMACS profile 5–7 or not provided: INTERMACS profile for 5 patients in the MOMENTUM trial axial-flow pump recipient group were not assessed.

### Percentage of real-world patients meeting trial exclusion criteria

**Fig 1:**
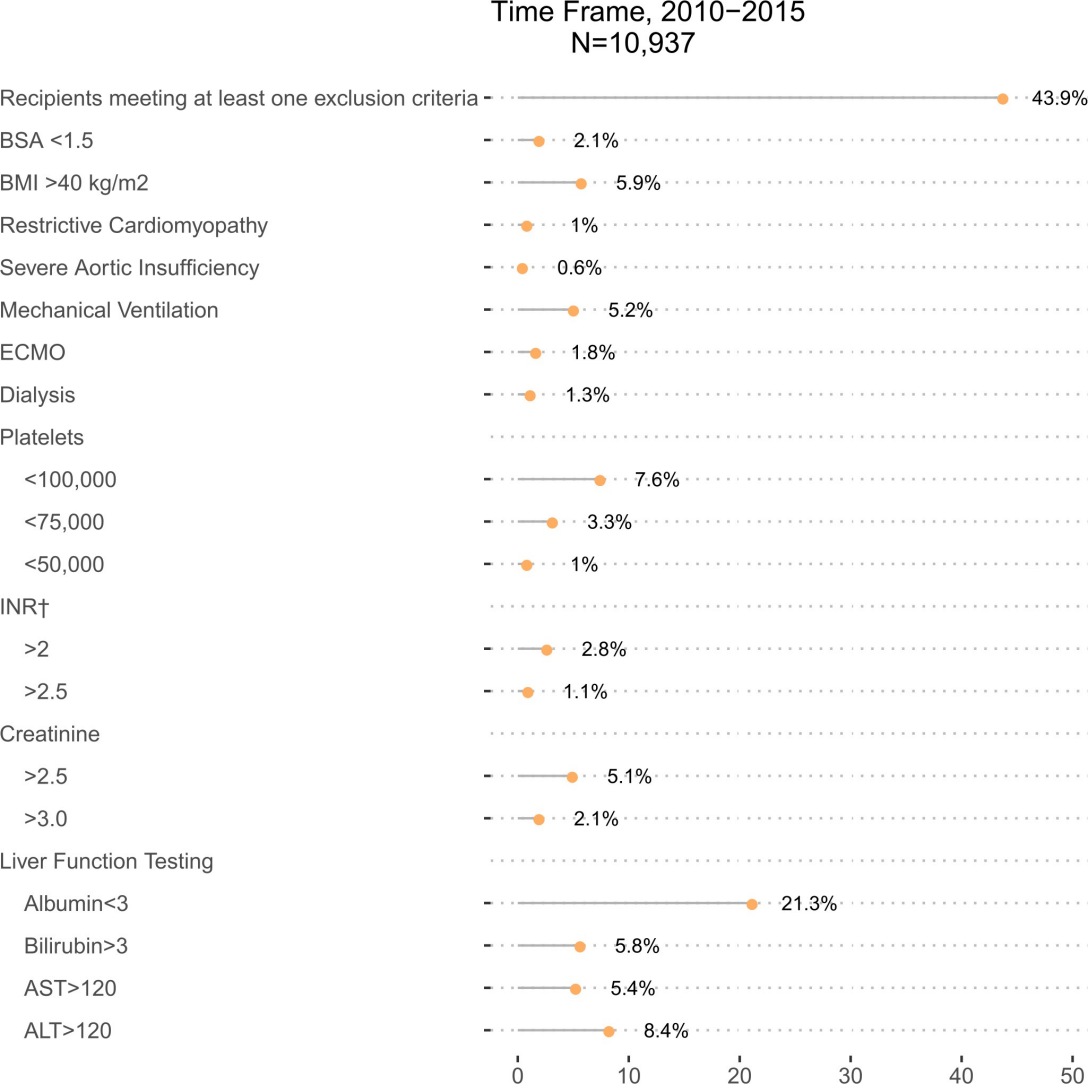
Percentages of axial flow LVAD recipients 2010–2015 meeting key clinical trial exclusion criteria. †Not on Warfarin.

The percentage of patients with axial flow LVADs in the INTERMACs database who met trial exclusion criteria are shown in **Fig 1**. In aggregate, 43.9% of patients in the INTERMACS database from 2010–2105 had at least 1 exclusion criterion noted in clinical trials. The most frequent clinical trial exclusion criteria present were hypoalbuminemia<3 g/dl (21.2%), BMI >40 kg/m^2^ (5.9%), thrombocytopenia<100,000/ul (7.6%), creatinine>2.5 mg/dl (5.1%), bilirubin>3 mg/dl (5.8%), elevated liver chemistries (ALT/AST; 5.4%/8.4%) and having required mechanical ventilation prior to implant (5.2%). However, proportions for patients meeting specific exclusion criteria were generally low, except in the case of the low albumin exclusion criteria, enforcement of which would exclude more than 10% of patients implanted with an LVAD in the real-world setting.

### Adverse clinical outcomes

Commonly encountered adverse events in the first 2 years after implant in the INTERMACS registry versus clinical trials are shown in **Table 2**. Of real-world patients who received an axial flow LVAD between 2010–2015, 26.1% died, 15.7% had a stroke, and 14.0% required pump reoperation. Stroke was more frequent in INTERMACS than in HM2 patients enrolled into MOMENTUM 3 but less frequent than HM2 patients enrolled in ENDURANCE. Death was more frequent among HM2 patients in ENDURANCE. Common adverse events included bleeding, infection, arrhythmia, right heart failure, and respiratory failure, whose frequencies are also shown in **Table 2**. LVAD recipients in INTERMACS who met trial exclusion criteria had higher rates of mortality, bleeding, arrhythmia, respiratory failure, hepatic dysfunction, and renal dysfunction over the 2 year follow up interval, although several of these absolute risk differences were small. Axial flow LVAD patients in the INTERMACS registry (2010–2015) appeared to have lower rates of most complications than those in the ENDURANCE and MOMENTUM 3 clinical trials, potentially due to differences in adjudication strategies.

**Table 2:** Outcomes at 2 years post implantation for axial flow LVADs placed 2010–2015 compared with published clinical trials.

Outcomes	HM II Recipients (MOMENTUM)*	INTERMACS	HM II Recipients (ENDURANCE)	*P*	INTERMACS with no exclusion criteria	INTERMACS with 1+ exclusion criteria	*P*
		Axial Flow LVADs					
	N = 505						
		N = 10,937	N = 149				
					N = 6,133	N = 4,804	
Primary endpoints							
Death	13.1^†^	26.1	32.2	<0.001	23.7	29.3	<0.001
Stroke^‡^	19.4	15.7	12.1	0.04	15.8	15.5	0.70
Pump reoperation	14.3^†^	14.0	16.2^†^	0.73	14.1	14.0	0.91
Adverse events							
LVAD Infection	19.4	17.0	15.4	0.32	16.9	17.0	0.89
Bleeding	55.0	45.6	60.4	<0.001	44.6	46.9	0.02
Right Heart Failure	28.3	24.8	26.8	0.18	24.1	25.6	0.07
LVAD Thrombosis	13.9	12.1	10.7	0.43	12.8	11.2	0.01
Arrhythmias	41.0	29.1	40.9	<0.001	28.3	30.1	0.04
Hepatic Dysfunction	5.3	5.8	8.1	0.46	4.8	7.2	<0.001
Respiratory Failure	19.4	20.4	25.5	0.26	17.7	23.9	<0.001
Renal Dysfunction	11.1	14.4	12.1	0.09	12.0	17.4	<0.001

*2.3% (4 patients) who did not receive the assigned implant and 1.7% (3 patients) who withdrew from the trial after implant were counted toward the primary outcome in the MOMENTUM trial.

^†^Primary endpoint of death and pump reoperation was reported with N = 512 in MOMENTUM and pump reoperation was reported with N = 148 in ENDURANCE.

^‡^Data on Rankin score were not available for enough stroke events to quantify severity of stroke. Here, stroke includes all strokes recorded as an adverse event. Sources and definitions in **S1 Table**.

### Real-world outcomes according to trial exclusion criteria

**Fig 2:**
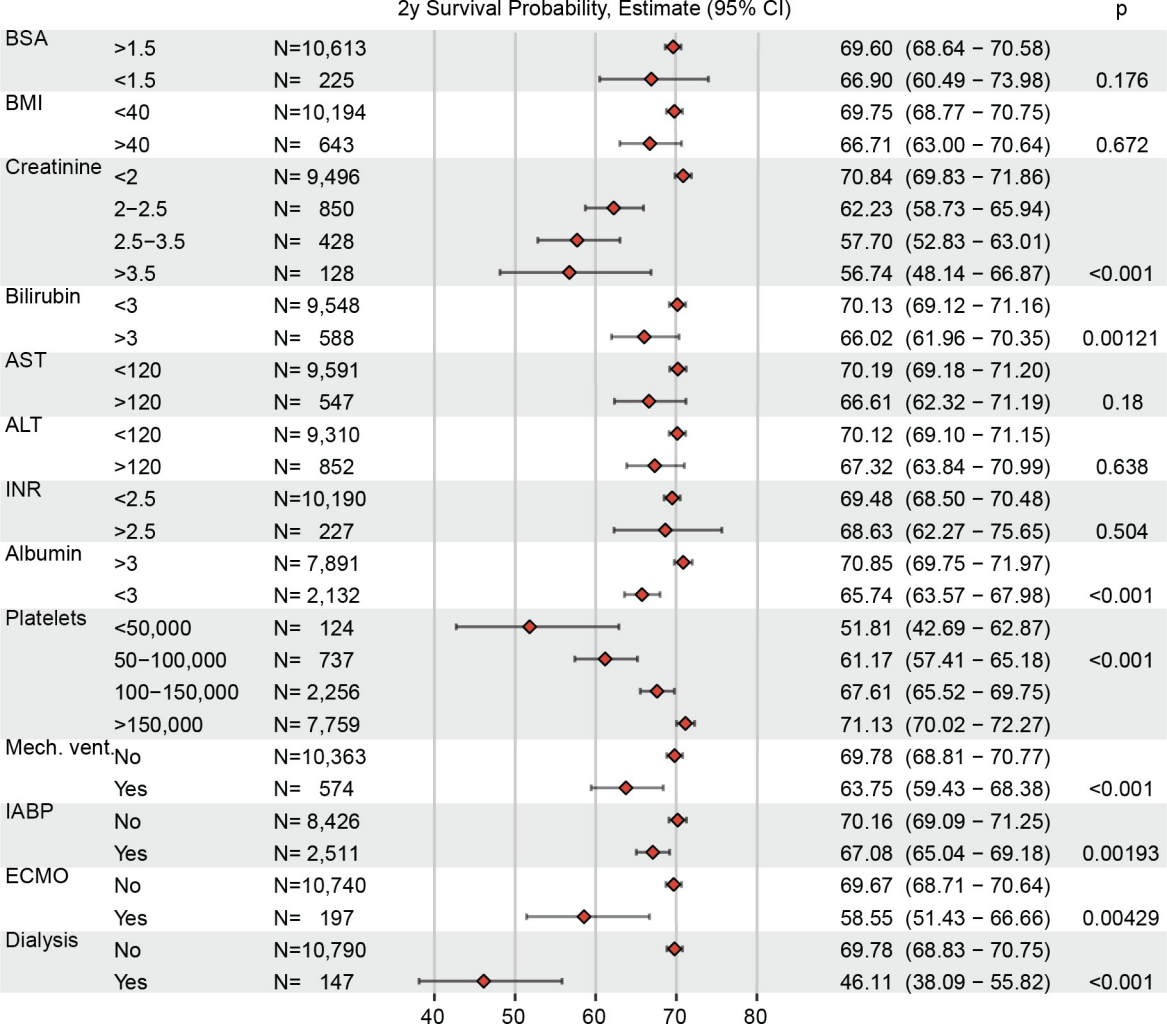
Survival estimates at 2 years according to key patient criteria among axial flow LVAD recipients enrolled in INTERMACS between 2010–2015. N shows number of patients in each sub-group. Points in the plot shows survival probability at 2y; error bars show 95% CI. Numerical estimate and 95% CI are shown with Log-rank test p-value.

**Fig 3:**
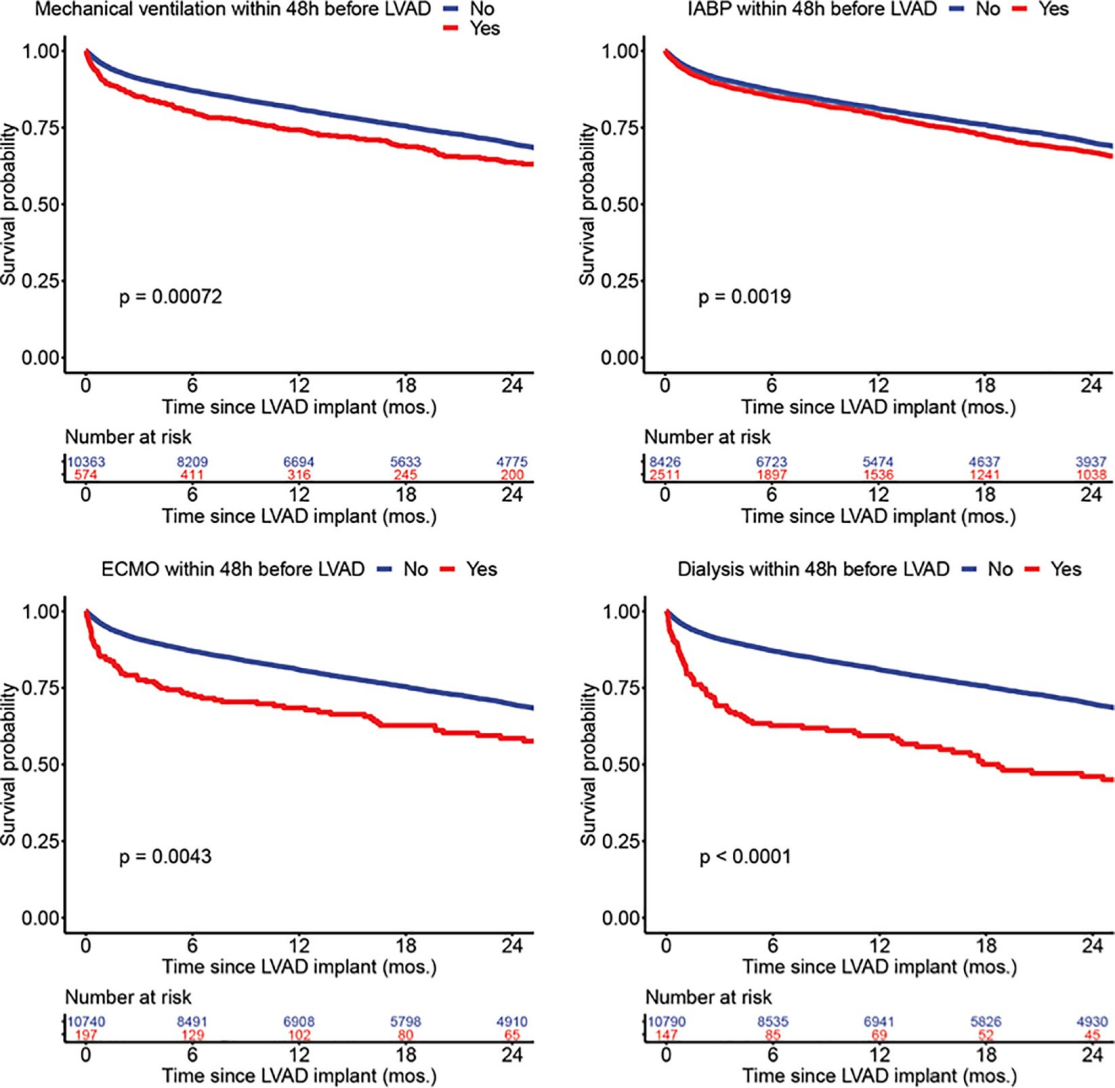
Kaplan-Meier survival estimates at 2 years for high risk interventions 48 hours prior to LVAD implantation among axial flow LVAD recipients enrolled in INTERMACS between 2010–2015.

**Fig 4:**
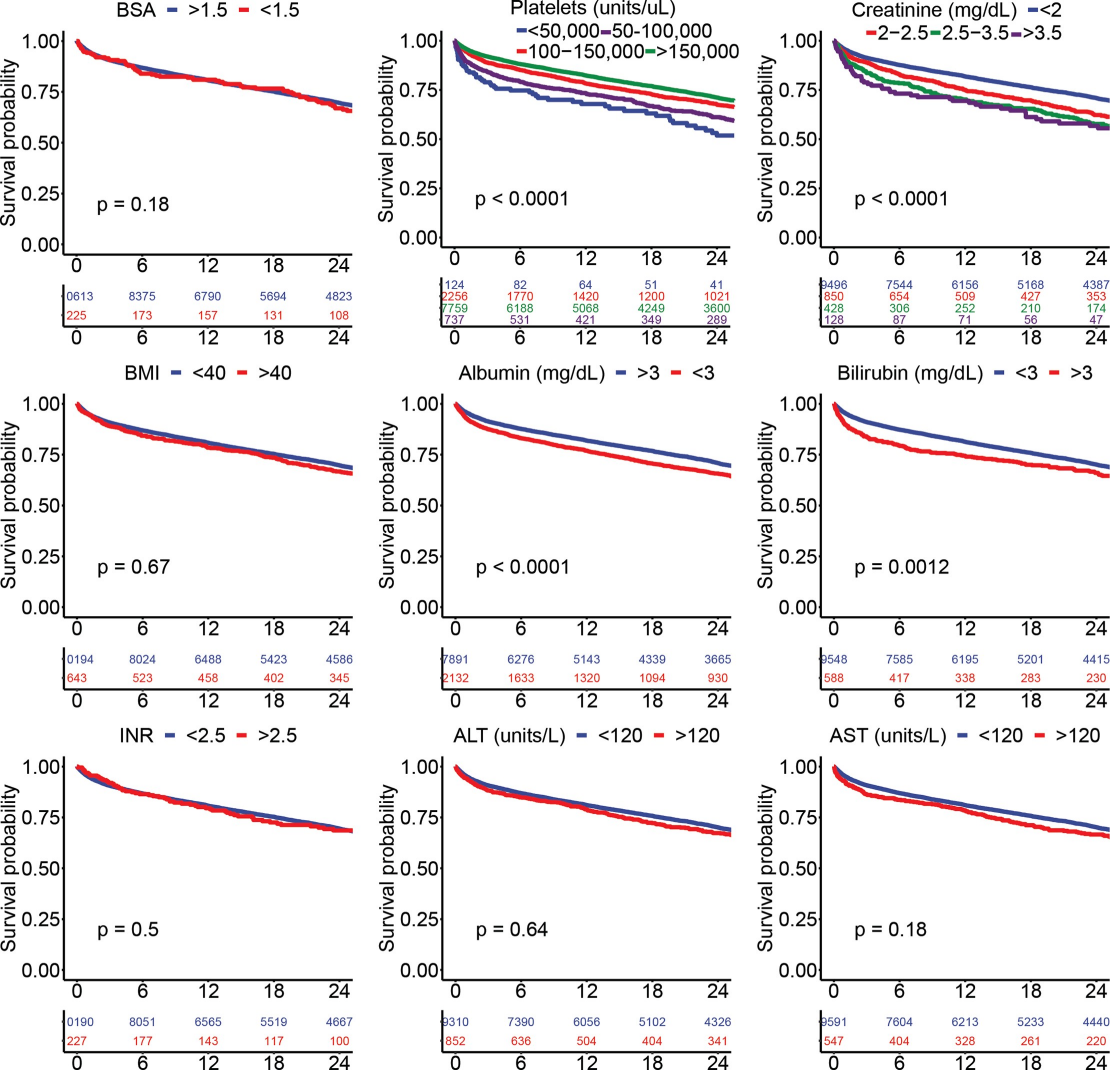
Kaplan-Meier survival estimates at 2 years according to clinical trial exclusion criteria among axial flow LVAD recipients enrolled in INTERMACS between 2010–2015.

Two-year survival estimates in INTERMACS patients according to the presence and absence of key patient factors used as clinical trial exclusion criteria are shown in **Fig 2**. As expected, patients with hypoalbuminemia <3 g/dl, higher baseline creatinine, thrombocytopenia, and hyperbilirubinemia had lower survival probabilities (*P*<0.001, all). Importantly, patients with BMI > 40 kg/m^2^, elevated LFTs, and increased INR did not have an increased risk of death. **Fig 3**demonstrates Kaplan-Meier survival estimates according to high risk interventions prior to LVAD surgery among INTERMACS patients. As shown, use of mechanical ventilation, IABP, ECMO, and dialysis in the 48 hours prior to LVAD placement were all associated with increased risk of death (*P*<0.001, all). **Fig 4**demonstrates Kaplan-Meier survival estimates according to key clinical trial exclusions, demonstrating that thrombocytopenia, increased creatinine, low albumin, and high bilirubin were associated with decreased survival at 2 years. In multivariate analysis accounting for all exclusion criteria shown in **S2 Table**, BMI > 40, albumin < 3, bilirubin > 3, higher creatinine, and hemodialysis within 48 hours of surgery were significantly associated with increased 2-year mortality (P<0.01, all).

**Fig 5:**
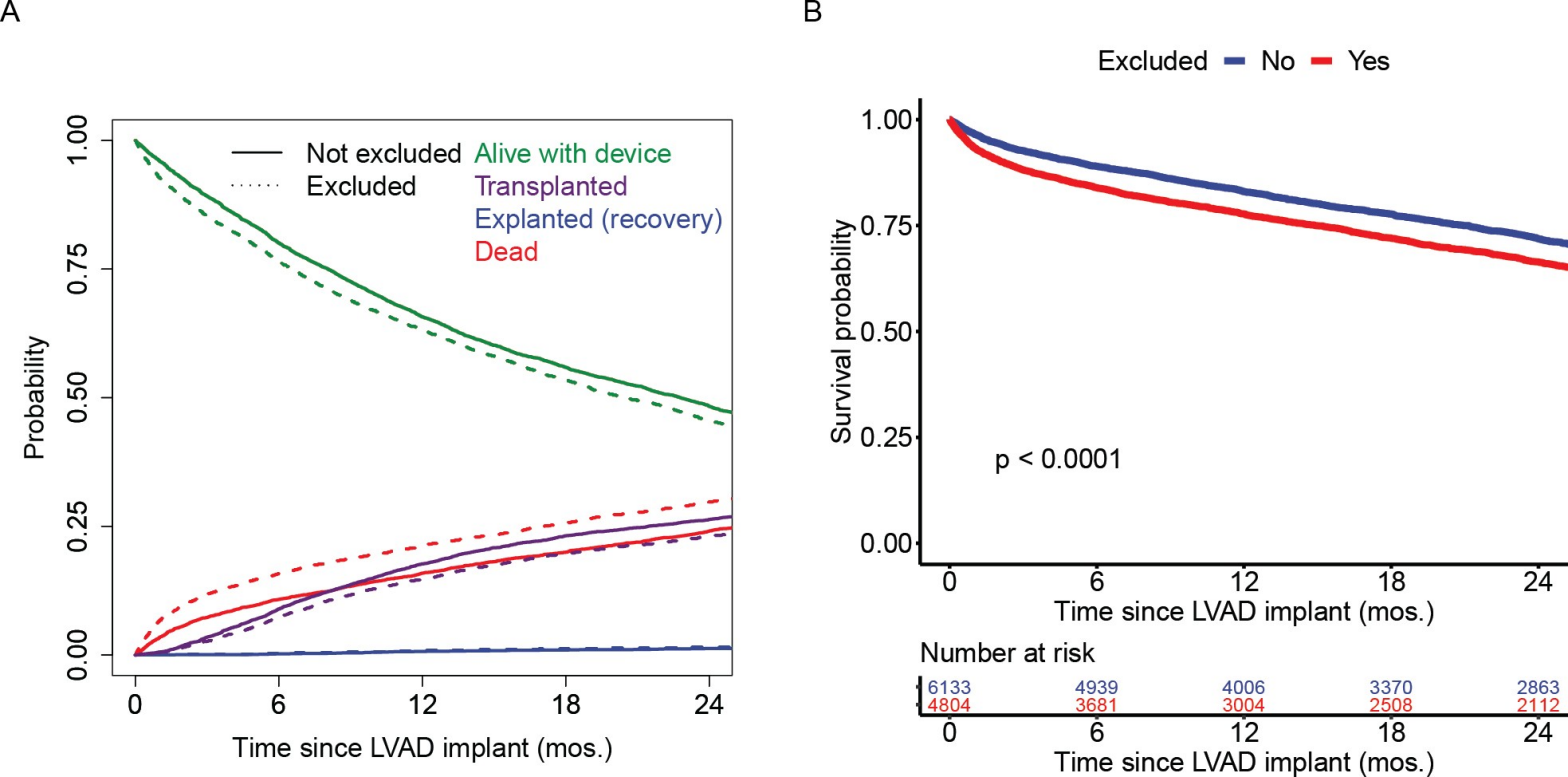
Competing risks of outcomes between real world LVAD patients who would and would not qualify for landmark clinical trials.

Finally, we examined risk of death between patients in INTERMACS who would versus would not have qualified for major clinical trials based on all exclusion criteria (**Fig 5**). As shown, the competing risks of outcomes did not differ substantially between patients who met and did not meet the exclusion criteria for clinical trials. Patients who would not have qualified for the clinical trials had an increased risk of death (*P*<0.001), but absolute rates of mortality did not appear to be dramatically different [2-year survival estimate: 66.4%, 95% CI (64.9–67.9%) versus. 71.9%, 95% CI, (70.6–73.1%)].

## Discussion

This study comparing differences in outcomes between patients who received axial flow LVADs as a part of two landmark clinical trials and those in an ongoing registry of patients led us to a few key insights about the clinical use of these devices. First, we found that the proportion of patients in INTERMACS who met individual specific clinical trial exclusion criteria was generally low (a majority less than 5%), with the exception of hypoalbuminemia. Despite this, 44% of patients in INTERMACS met at least one clinical trial exclusion criteria. Second, key adverse clinical outcomes were not substantially varied between clinical trials and the registry, with demonstration of better outcomes in the more recent clinical trial (MOMENTEM 3). Third, as expected, patients who underwent high risk interventions prior to implantation such as intubation, hemodialysis, and ECMO had an increased risk of death; however, this risk appeared lower than what has been reported in patients with stage D HF [8]. Finally, patients who would not have qualified for the clinical trials had an increased risk of death, but absolute rates of mortality did not appear to be dramatically different. In their entirety, these data suggest that most exclusion criteria used in LVAD clinical trials did not afford a substantially greater risk to patients in the real-world setting. However, in the relatively rare cases of severe thrombocytopenia, advanced renal disease, respiratory failure, and need for dialysis, the risk of death after device implantation was substantially higher than in the overall cohort.

Despite initial enthusiasm for LVAD therapy in end stage HF patients, and dramatic improvements in clinical outcomes, clinical use has plateaued at levels far less than previously forecasted [9]. Our findings offer a potential explanation for this finding—unlike in the case of other therapeutics where real-world use is expanded beyond the population studied in the clinical trial, treatment with LVADs appears to be quite conservative considering the number of patients who are inferred to benefit from this intervention [10–12]. The causes of this behavior are unclear and might result from several factors such as patient preferences, lack of knowledge among most physicians about the therapy, and misunderstandings regarding candidacy [13]. Our data suggest that whereas patients who did not qualify for landmark clinical trials might not obtain the degree of survival benefit seen in the clinical trial setting, the absolute rates of mortality are not dramatically different.

Our study does demonstrate that a subset of patients undergoing LVAD implantation—namely those with severe thrombocytopenia, advanced renal disease, respiratory failure, and need for dialysis—have a high risk of mortality post implantation. Whereas we can only speculate about what the alternative might have been if an LVAD had not been offered, it is important to carefully weigh the risks and the benefits in such cases. In this regard, a fair amount of work has been done in the realm of cost effectiveness and changes in quality of life with LVAD therapy among a wider population of end stage HF patients; it might be an opportune time to examine these questions among patients whose comorbidities cause them to have higher than usual rates of adverse events, prolonged hospitalizations, and survival without a meaningful quality of life [14–16].

Several limitations of this analysis require consideration. First, we only included patients who received axial flow devices after 2010 to minimize confounding by device type—these are by far the most common devices in the INTERMACS dataset. It is likely that inclusion of other, especially newer devices, would have shown a different association with outcomes. Second, only data collected per registry protocol was available with substantial missingness of several variables, leading to unmeasured confounding and less than ideal granularity in our analysis. The largest proportions of missing data were for albumin, which was missing for 914 LVAD recipients or 8% of the study sample, bilirubin, which was missing for 801 recipient or 7% of the sample, and INR, which was missing for 520 recipients or 5% of the sample. Third, information on landmark clinical trials was derived from published information rather than the source data, limiting a more comprehensive comparison with registry patients. Finally, it is very likely that the methods for adjudication of adverse events other than death very between clinical trials and INTERMACS, leading to misclassification in some cases. In summary, we considered this analysis to examine and explain trends in the real-world usage of LVADs rather than focus on prognostic implications of various risk factors.

By examining real-world patients who received axial flow LVADs, we found that those who would not have qualified for the landmark clinical trials had an increased risk of death, but their absolute rates of mortality did not appear to be dramatically different. In rare cases of high-risk patients with respiratory failure, thrombocytopenia, renal failure, and need for ECMO, risk of mortality was significantly higher. In their entirety, these data suggest that most exclusion criteria used in LVAD clinical trials did not afford a substantially greater risk to patients in the real-world setting.

## Supporting information

S1 Table Sources for numbers reported in main text. (DOCX)

S2 Table Multivariate logistic regression for 2-year mortality among recipients of axial flow LVADs 2010–2015. (DOCX)
