# Supplementary material for: Clinical implications of differences between real world and clinical trial usage of left ventricular assist devices for end stage heart failure
Source: PLoS One. 2020 Dec 3;15(12):e0242928. doi: 10.1371/journal.pone.0242928 (PMC7714148; doi:10.1371/journal.pone.0242928)
Supplement: S1 Table — (DOCX) [file pone.0242928.s001.docx]

**S1 Table.** Sources for numbers reported in main text.

| **Outcomes** | **HM II Recipients (MOMENTUM)** | **INTERMACS**  **Axial Flow LVADs**^⁑^ | **HM II Recipients (ENDURANCE)** |
| --- | --- | --- | --- |
| Death | Table 2 | Patients: DEAD_PT | Table 3 |
| Stroke^‡^ | Table S6 | Events: AE_NEURO_STROKE | Table 3 |
| Pump reoperation | Table 2 | Events: EXPLANT_*_DEV_*, EXPLANT_TXPL_THROM | Table 2 |
| LVAD Infection^†^ | Table S6 | Events: INFECT_LOC_PUMP_DRIVELINE | Table 3 |
| Bleeding | Table S6 | Events: BLEEDING | Table 3 |
| Right Heart Failure | Table S6 | Follow-up: RHF_FLG | Table 3 |
| LVAD Thrombosis | Table S6 | Events: AE_DEV_THR_EVNT, EXPLANT_*_DEV_THR_*, EXPLANT_TXPL_THROM | Table 3 |
| Arrhythmias | Table S6 | Events: CARD_ARRYTHMIA | Table 3 |
| Hepatic Dysfunction | Table S6 | Events: HEPATIC | Table 3 |
| Respiratory Failure | Table S6 | Events: RESP_FAIL | Table 3 |
| Renal Dysfunction | Table S6 | Events: RENAL_DYS | Table 3 |

^⁑^INTERMACS variables used for Table 2 (denoted as dataset: variables) with INT_DPT, INT_EVT, and INT_AFOL (for the patients, events, and follow-up datasets, respectively) indicating first-incidence of outcome within 2y post-implant. ^†^Data on Rankin score were not available for enough stroke events to quantify severity of stroke. As such, the number reported in the main text reports incidence of any stroke. ^‡^Driveline infections were reported since this definition was consistent between trials while definitions of other infection locations differed. *Variables used to define pump reoperation and LVAD thrombosis in INTERMACS included explant due to exchange, no new device, and device turned off for malfunction or thrombosis.
