# Supplementary material for: Clinical implications of differences between real world and clinical trial usage of left ventricular assist devices for end stage heart failure
Source: PLoS One. 2020 Dec 3;15(12):e0242928. doi: 10.1371/journal.pone.0242928 (PMC7714148; doi:10.1371/journal.pone.0242928)
Supplement: S2 Table — (DOCX) [file pone.0242928.s002.docx]

**S2 Table.** Multivariate logistic regression for 2-year mortality among recipients of axial flow LVADs 2010-2015.

| Predictor | OR | 95% CI | p |
| --- | --- | --- | --- |
| BSA < 1.5 | 1.11 | 0.90-1.37 | 0.36 |
| BMI > 40 | 1.30 | 1.13-1.50 | <0.001 |
| Creatinine < 2  Creatinine 2-2.5  Creatinine 2.5-3  Creatinine 3-3.5 | Ref  1.26  1.41  1.11 | 1.12-1.41  1.41-1.21  0.83-1.49 | <0.001  <0.001  0.50 |
| Bilirubin > 3 | 1.21 | 1.06-1.40 | 0.007 |
| AST > 120 | 1.05 | 0.87-1.25 | 0.63 |
| ALT > 120 | 0.92 | 0.79-1.06 | 0.25 |
| INR > 2.5 | 0.89 | 0.71-1.12 | 0.31 |
| Albumin < 3 | 1.12 | 1.03-1.21 | 0.008 |
| Platelets <50,000  Platelets 50-100,000  Platelets 100-150,000  Platelets > 150,000 | Ref |  |  |
| Mechanical ventilation | 1.08 | 0.92-1.27 | 0.35 |
| IABP | 1.06 | 0.98-1.14 | 0.17 |
| ECMO | 1.18 | 0.92-1.52 | 0.19 |
| Dialysis | 1.58 | 1.23-2.02 | <0.001 |
